# A comparison of gonadotropin-releasing hormone and human chorionic gonadotropin in dairy cows with ovarian follicular cysts

**DOI:** 10.18849/ve.v7i2.509

**Published:** 2022-04-21

**Authors:** Kathryn Kesler+, Grace Longcore+, Alex Russell+

**Keywords:** BOVINE, CYSTIC OVARIAN DISEASE, CYSTIC OVARIAN FOLLICLES, DAIRY CATTLE, GNRH, HCG, OVARIAN CYST, REPRODUCTION, TREATMENT

## Abstract

PICO question

In adult dairy cows with ovarian follicular cysts, does treatment with gonadotropin-releasing hormone (GnRH) compared to human chorionic gonadotropin (hCG) result in a more rapid return to cyclicity?

Clinical bottom line

Category of research question

Treatment

The number and type of study designs reviewed

The publications consisted of six non-blinded randomised comparative or controlled trials

Strength of evidence

Weak

Outcomes reported

Recovery time, clinical cure, and interval to conception were consistently evaluated. Many studies also evaluated other fertility parameters such as first estrus or first treatment conception, overall pregnancy and conception risks, and breedings per conception

Conclusion

At this time, there is insufficient evidence to suggest whether GnRH or hCG is more efficacious for treating ovarian follicular cysts in dairy cattle. Ultimately, further research is essential to elucidate which treatment results in a more rapid return to cyclicity for dairy cattle afflicted with cystic ovarian follicles

## How to apply this evidence in practice

The application of evidence into practice should take into account multiple factors, not limited to: individual clinical expertise, patient’s circumstances and owners’ values, country, location or clinic where you work, the individual case in front of you, the availability of therapies and resources.

Knowledge Summaries are a resource to help reinforce or inform decision making. They do not override the responsibility or judgement of the practitioner to do what is best for the animal in their care.

## Clinical scenario

Cystic ovarian disease (COD) has been reported to occur at an incidence of between 6% and 30% of dairy cattle and is generally accepted as a common cause of reproductive failure in the dairy cow (Silvia et al., 2002; Ono et al., 2018; De Rensis et al., 2008; and Kesler & Garverick, 1982). Cystic ovarian disease is the presence of an anovulatory follicle ≥25 mm in diameter that persists for a minimum of 10 days in the absence of a functional corpus luteum (CL) (Ono et al., 2018; De Rensis et al., 2008; Garverick, 1997; and Kesler & Garverick, 1982). There are predominantly two classifications of ovarian cysts, follicular and luteal, distinguishable by the presence or absence of luteal tissue and circulating progesterone concentrations (Jeengar et al., 2014).  Cystic ovarian follicles have been reported to extend the calving interval by 20–64 days and, therefore, are of great economic importance to dairy producers (Silvia et al., 2002; and De Rensis et al., 2008). Moreover, the economic impact that results from the labour required for treatment and exogenous hormonal therapy for return to cyclicity cannot be overlooked. Many reports utilise exogenous hormones such as gonadotropin-releasing hormone (GnRH) and human chorionic gonadotropin (hCG) for cyst luteinisation (Gundling et al., 2015 Rizzo et al., 2011; Kawate et al., 2011; and Nakao et al., 1980). This Knowledge Summary will address the question about which luteinising hormone results in a faster return to cyclicity.

You are a veterinarian performing routine herd health utilising ultrasound on a commercial dairy in the USA. The producer mentions that they are experiencing a high number of cystic cows on herd check. This producer asks you for recommendations regarding treating these cows; specifically, they want to get these cows cycling as quickly as possible.

## The evidence

The literature search elicited six publications that met our criteria for inclusion. The six publications consisted of five randomised non-blinded comparative or controlled trials of level 3 (lower quality controlled trials [Howick et al., 2011]), (Taktaz et al., 2015; Mollo et al., 2012; Garverick et al., 1976; Elmore et al., 1975; and Nakao et al., 1992) and one randomised controlled trial of level 2 (well-designed controlled trial [Howick et al., 2011]), (Verma & Dabas, 1994). Additionally, many articles did not compare GnRH and hCG treatments directly or did not utilise time as a metric for evaluation and thus were excluded from this Knowledge Summary.

Dairy cattle were used in all of the evaluated articles, with 1/6 studies involving Holsteins, 2/6 studies involving crossbred cattle, 1/6 studies involving Friesians, and 2/6 studies involving Guernsey and Holstein cattle. All articles involved classification of ovarian follicular cyst size and persistence of at least one week; three articles evaluated cattle for the presence of luteal tissue either utilising ultrasound (Taktaz et al., 2015) or progesterone assays (Garverick et al., 1976; and Nakao et al., 1992). Cystic ovarian follicles were treated in all of the articles with GnRH and hCG therapy. The number of cows in each study ranged from relatively small studies with 20 cows to moderately sized comparisons, with 150 cows. The number of cows allocated for each treatment was highly variable per study ranging from 3–70 animals. Three studies had large treatment groups (30–70 animals per treatment) (Mollo et al., 2012; Taktaz et al., 2015; and Elmore et al., 1975), 1/6 studies had moderately sized treatment groups (17–18 animals per treatment) (Nakao et al., 1992), and 2/6 had very small treatment groups (10 animals per treatment) (Verma & Dabas,1994; and Garverick et al., 1976). Clinical cure (return to oestrus or cyst luteinisation), first oestrus conception, and interval to conception were consistently evaluated in the vast majority of studies. Additionally, many studies evaluated parameters such as recovery time, overall conception risk, pregnancy risk, breedings per conception, and interval to insemination.

In studies utilising a negative control group with no treatment, there was no significant difference in clinical response or subsequent fertility between GnRH and hCG, while both therapies outperformed the control group (Verma & Dabas, 1994). A higher percentage of animals treated with GnRH responded after one treatment than hCG; however, there were no significant differences in the number of animals that responded to either treatment or how quickly they responded to each treatment (Verma & Dabas, 1994). In studies that evaluated clinical cure as a return to oestrus, there were mixed results, with two studies showing hCG was more effective (Mollo et al., 2012; and Garverick et al., 1976) and two studies showing GnRH had more favourable results (Verma & Dabas, 1994; and Elmore et al., 1975). One study evaluated clinical cure as luteinisation of the cystic ovarian follicle (COF), and they found that hCG luteinised (14/17, 82.3%) 21.2% more cows than GnRH (11/18, 61.1%) (Nakao et al., 1992). Articles that studied interval to conception resulted in contradictory evidence, with two studies finding hCG treatment protocols required fewer days to conception (Taktaz et al., 2015; and Nakao et al., 1992) and two studies finding that GnRH had a shorter interval to conception (Garverick et al., 1976; and Elmore et al., 1975). Five out of six studies evaluated recovery time as days to first oestrus, and while there were no biologically or statistically significant differences, the majority of articles found that hCG required fewer days to first oestrus than GnRH treatment (Taktaz et al., 2015; Mollo et al., 2012; and Elmore et al., 1975), although one article had conflicting results (Garverick et al., 1976). Verma & Dabas (1994) listed their responses as a range of days to first oestrus for each treatment, and there was significant overlap between the GnRH treatment (15–30 days) and hCG treatment (17–31 days). Henceforth, there is still much research that needs to be conducted as to which treatment induces a more rapid return to cyclicity in dairy cattle with COD.

Many studies evaluated additional fertility parameters such as conception after first treatment or artificial insemination (AI), overall pregnancy or conception risks, and breedings per conception. Studies that evaluated conception after the first treatment or first AI had mixed results, with two articles indicating that hCG resulted in higher conception (Taktaz et al., 2015; and Mollo et al., 2012) and three articles suggesting GnRH had higher conception risks (Verma & Dabas, 1994; Elmore et al., 1975; and Nakao et al., 1992). When overall pregnancy risk was evaluated, Taktaz et al. (2015) at 70 days post treatment and Mollo et al. (2012) at 7 weeks post AI, found that hCG resulted in higher pregnancy risks; however, at 100 days, the Taktaz et al. (2015) group found that GnRH had a higher pregnancy risk. Moreover, when overall conception risk was evaluated, two groups found hCG to be more favourable (Elmore et al., 1975; and Nakao et al., 1992), while one group found GnRH to result in a higher conception risk (Garverik et al., 1976). Lastly, two studies evaluated the number of breedings per conception, while insignificant, both found that hCG treatment required more breedings per conception than GnRH treatments (Garverick et al., 1976; and Elmore et al., 1975).

## Summary of the evidence

**Mollo et al. (2012):** 

Population:	Cystic Friesian cows diagnosed with follicular structures > 25 mm, persistent for > 7 days, in the absence of a corpus luteum.
Sample size:	n = 150.
Intervention details:	Group 1: 20 ug gonadotropin-releasing hormone (GnRH) (n = 70). Group 2: 3,000 international units (IU) human chorionic gonadotropin (hCG) (n = 50). Group 3: Progesterone releasing intravaginal device for 10 days (n = 30). All animals were checked twice daily for oestrus. Cows were artificially inseminated 12 hours after oestrus detection.
Study design:	Prospective non-blinded, randomised controlled trial.
Outcome studied:	Cure rate: The ratio between cows in oestrus within 30 days post-treatment and treated cows. Recovery time: The time between treatment and standing oestrus. First oestrus conception rate (pregnant cows / inseminated cows). Overall pregnancy rate (pregnant cows / treated cows).
Main Findings (relevant to PICO question)	No statistical differences were found between cure rates, conception rates, and overall pregnancy rates. Cure rates* (%): 64.3, 66.0, and 63.3; Conception rates* (%): 45.2, 47.8, and 46.2; Pregnancy rates* (%): 20.0, 22.0, and 20.0; Recovery time* (days ± confidence interval [CI]) 17.9 ± 3.1, 17.7 ± 3.2, 19.7 ± 4.7. *For groups 1,2, and 3 respectively.
Limitations:	Long study window (4 years): Weather variation; Protocol drift. Animals were only observed twice daily for oestrus detection.

**Taktaz et al. (2015):** 

Population:	Lactating Holstein dairy cattle (35–120 days in milk), diagnosed with ovarian cysts > 25 mm and ultrasonically classified as follicular or luteal.
Sample size:	n = 144.
Intervention details:	Group 1 (n = 47): Day 0: 0.02 mg gonadotropin-releasing hormone (GnRH) (Buserelin); Day 10: 500 ug prostaglandin F_2α_ (PGF_2α_) (Cloprostenol). Group 2 (n = 47): Day 0: 0.02 mg GnRH (Buserelin) and 500 ug PGF_2α_ (Cloprostenol): Day 10: 500 ug PGF_2α_ (Cloprostenol). Group 3 (n=50): Day 0: 1500 IU human chorionic gonadotropin (hCG) (Chorulon);and 500 ug PGF_2α_ (Cloprostenol); Day 10: 500 ug PGF_2α_ (Cloprostenol). All cattle were artificially inseminated 12 hours after the onset of oestrus.
Study design:	Prospective non-blinded, randomised controlled trial.
Outcome studied:	Recovery time. Interval to conception. Conception rate at first artificial insemination (AI). Pregnancy rate by day 70 and day 100.
Main Findings (relevant to PICO question)	No statistical differences were found between groups for recovery time, interval to conception, conception rate at first AI, and pregnancy rate by days 70 and 100: Recovery time* (days ± confidence interval [CI]): 18.1 ± 1.5, 16.7 ± 1.5, 15.4 ± 1.4; Interval to conception* (days ± CI): 55.9 ± 8.2, 76.1 ± 13.3, 57 ± 7.3; Conception rate* (%): 45.4, 34.5, 44.4; Pregnancy rate by day 70* (%): 78.8, 69, 69.4; Pregnancy rate by day 100* (%): 81.8, 75.9, 75.0. *For groups 1, 2 and 3 respectively with follicular cysts.
Limitations:	The majority of cysts diagnosed in this study were follicular cysts (98/144, 68%), with substantially less luteal cysts (46/144, 32%); PGF_2α_ has been reported to be less efficacious for follicular cysts. This may have impacted the response to treatment for groups who received PGF_2α_ on Day 0 (groups 2 and 3).

**Verma & Dabas (1994):** 

Population:	Crossbred cattle with confirmed cystic ovarian disease (semiweekly rectal palpation for 1 month) with a history of irregular oestrus and persistently enlarged cysts on ovaries.
Sample size:	n = 21.
Intervention details:	Gonadotropin-releasing hormone (GnRH) group (n = 8): 200 mcg GnRH (Receptal). Human chorionic gonadotropin (hCG) group (n = 10): 3000 IU hCG (Chorulon). Control group (n = 3): Sterile saline. All cattle were evaluated behaviourally and via transrectal palpation starting 3 days post-treatment for response to therapy. All animals with a positive response to therapy were subsequently artificially inseminated.
Study design:	Prospective non-blinded, randomised controlled trial.
Outcome studied:	A positive response to therapy was recorded if normal oestrus cycle followed or subsequent conception. A negative response to therapy was recorded if no change in cyst structure was observed.
Main Findings (relevant to PICO question)	5/10 (50%) of animals treated with hCG showed normal oestrus within 17–31 days: All five animals were artificially inseminated and successfully conceived. 5/8 (62.5%) of animals treated with GnRH showed normal oestrus within 15–30 days: All five animals were artificially inseminated and successfully conceived. No change was observed in the control group.
Limitations:	Small sample size. Cysts were classified as follicular cysts based on rectal palpation and irregular oestrus, which is less accurate than progesterone assays or ultrasound classification. Confidence intervals were unavailable.

**Elmore et al. (1975):** 

Population:	69 Holstein, 15 Guernsey cattle diagnosed with at least one ovarian cyst 2.5 cm or larger via rectal palpation.
Sample size:	n = 84.
Intervention details:	Gonadotropin-releasing hormone (GnRH) group (n = 43): 100 mcg GnRH. human chorionic gonadotropin (hCG) group (n = 41): 10,000 IU hCG. All cattle had their ovaries examined via rectal palpation 14 days post-treatment for response to therapy; if a change was noted or suspected animals were reexamined within two weeks.
Study design:	Prospective non-blinded, randomised controlled trial.
Outcome studied:	A positive outcome was recorded if subsequent oestrus behaviour and fertility were observed. A negative outcome was recorded if no changes in the site, size, or character of the cyst(s) were observed.
Main Findings (relevant to PICO question)	No statically significant differences were found between groups. More cows treated with GnRH had a positive outcome (34/43, 79%) than those treated with hCG (28/41, 68%). Cows treated with hCG had a faster onset of oestrus at 19.7 ± 3.4 (days ± CI) versus cows treated with GnRH 21.2 ± 3.3 (days ± CI). Cows treated with GnRH required fewer breedings (1.4 ± 0.7 per conception) and fewer days to conception (37.4 ± 4.9) as compared to those treated with hCG (1.8 ± 0.9 and 47.8 ± 5.6, respectively).
Limitations:	No attempt was made to differentiate between follicular and luteal cysts.

**Garverick et al. (1976):** 

Population:	Holstein and Guernsey cattle were diagnosed with at least one smooth, fluctuant, round structure 2.5 cm in diameter minimally on one or both ovaries.
Sample size:	n = 20.
Intervention details:	Gonadotropin-releasing hormone (GnRH) group (n = 10): 100 mcg GnRH. Human chorionic gonadotropin (hCG) group (n=10): 10,000 IU hCG. All cattle were observed for oestrus behaviour, blood samples were collected, and rectal palpation was performed every 4 days post-treatment until oestrus for up to 30 days. Blood samples were collected, and rectal palpations were performed on days 0, 1, 5, 9, and 13 post-oestrus.
Study design:	Prospective non-blinded, randomised controlled trial.
Outcome studied:	A positive outcome was recorded if the animal established a normal oestrus cycle or conceived. A negative outcome was recorded if no change was observed in the character, size, or location of the cyst within a 2–4 week period.
Main Findings (relevant to PICO question)	Differences in clinical response and subsequent fertility between GnRH and hCG treated cattle were not significantly different. More cattle positively responded to hCG treatment (9/10, 90%) than GnRH treatment (8/10, 80%). More days from treatment to first oestrus were seen with hCG treatment (20.5 ± 0.6 days) than GnRH treatment (17.8 ± 2.4 days). Cattle treated with hCG required more services per conception (3.5 ± 0.6) and had more days from treatment to conception (91 ± 23 days) than GnRH treated cattle (2.7 ± 0.2 and 59.7 ± 9 days, respectively).
Limitations:	Small sample size. Changes in ovarian structures were detected via rectal palpation; ultrasound is more accurate for detecting changes in ovarian structures.

**Nakao et al. (1992):** 

Population:	Crossbred dairy cattle were diagnosed with persistent ovarian cysts at least 2.5 cm in diameter (two successive rectal palpations 7 days apart) and no functional luteal tissue as confirmed by retrospective milk progesterone analysis.
Sample size:	n = 57, post progesterone exclusion n = 35.
Intervention details:	Gonadotropin-releasing hormone (GnRH) group (n = 18): 20 mcg GnRH (Buserelin). Human chorionic gonadotropin (hCG) group (n=17): Day 0: 10,000 IU hCG (Gestron). All cattle were transrectally palpated 10–14 days post-treatment; animals with luteinised cysts were artificially inseminated when they came into oestrus.
Study design:	Prospective non-blinded, randomised controlled trial.
Outcome studied:	Milk progesterone concentration indicative of luteinisation. Oestrus behaviour. Conception following artificial insemination.
Main Findings (relevant to PICO question)	More cattle treated with hCG (14/17, 82.3%) successfully responded to treatment than cattle treated with GnRH (11/18, 61.1%). There were more days from treatment to luteinisation with hCG treatment (5 ± 2, days ± standard deviation (SD)) than GnRH treatment (4 ± 2, days ± SD). There were more days from treatment to conception with GnRH treatment (36 ± 14 days) than hCG treatment (31 ± 7 days). More cattle conceived after the first treatment with the GnRH treatment (7/18, 38.9%) than hCG treatment (5/17, 29.4% cows). Days from treatment to conception were equivocal, 42 ± 18 days regardless of treatment.
Limitations:	Animals with luteal cysts were retrospectively excluded utilising a progesterone assay; if this would have been performed before enrollment or ultrasound was utilised in conjunction, the authors may have been able to increase enrollment.

## Appraisal, application and reflection

Ovarian follicular cysts are a common reproductive problem in the dairy industry, affecting approximately 6–30% of dairy cows (Silvia et al., 2002; Ono et al., 2018; De Rensis et al., 2008). However, the incidence of follicular cysts is likely underestimated, as 60% of the cysts that form before the first ovulation after freshening resolve without intervention (Ijaz et al., 1987; Jeengar et al., 2014; Kesler & Garverick, 1982). An ovarian follicular cyst has been described as a follicle that is 25 mm or larger in diameter and is persistent for at least 10 days in the absence of a CL (Garverick, 1997; Ono et al., 2018; De Rensis et al., 2008; Kesler and Garverick, 1982). Follicular cysts have been shown to add approximately 20 to 64 days to a dairy cow's open period (Silvia et al., 2002; De Rensis et al., 2008). Resulting in a significant negative economic impact for dairy producers, as a delay in conception leads to decreased milk yield, and resolution of the cyst often requires the expense of veterinary treatment.

The cause of ovarian follicular cysts is currently unknown (Vanholder et al., 2006, Amweg et al., 2013). There are several different treatment options for ovarian cysts, including manual rupture, aspiration, and hormonal injection.  Exogenous hormones are the most commonly used and successful form of treatment (Gundling et al., 2015 Rizzo et al., 2011, Kawate et al., 2011). Recently, manual rupture has become controversial due to results indicating that it may cause ovarian trauma that leads to the development of harmful haemorrhage and adhesions that can reduce fertility (Jeengar et al., 2014). Many reports argue the beneficial effects of gonadotropin-releasing hormone (GnRH) and human chorionic gonadotropin (hCG) for treating ovarian luteal and follicular cysts (Taktaz et al., 2015). In addition, prostaglandin-F_2α_ (PGF_2α_) has been shown to be effective against luteal cysts but not for follicular cysts. It is difficult to accurately determine whether a cyst is follicular or luteal by rectal palpation, and while accuracy is improved via ultrasound, progesterone assays remain the most definitive way of differentiating luteal and follicular cysts (Taktaz et al., 2015; Farin et al., 1992; Jeengar et al., 2014). Given the difficulty of distinguishing luteal and follicular cysts, Taktaz et al. (2015) suggested utilising a combination of PGF_2α_ and either GnRH or hCG to treat COD. However, Taktaz et al. (2015) did not find this combination therapy beneficial in shortening recovery time, interval to conception, conception risk, or pregnancy risk. Determining the most effective treatment method for ovarian follicular cysts is of utmost importance to dairy producers and veterinarians. The purpose of this Knowledge Summary is to evaluate the evidence comparing the length of time to return to ovarian cyclicity with the treatment of either GnRH or hCG.

The literature review resulted in six studies that met the inclusion criteria. Cystic ovarian follicles were identified utilising transrectal ultrasound or transrectal palpation. Mollo et al. (2012) evaluated recovery time, defined as the number of days between treatment and standing oestrus. Cattle were treated with GnRH (20 µg), hCG (3000 IU), or progesterone (1.55 g progesterone releasing intravaginal device for 10 days). However, the hCG group outperformed alternative treatments in every category (cure risk, pregnancy risk, and recovery time); no statistical or biologically significant differences were detected (Mollo et al., 2012). These results are further supported by Taktaz et al. (2015), who reported an insignificant 1.6 day reduction in recovery time with hCG (1500 IU) compared to conventional treatment (GnRH analogue) in cows with follicular cysts. However, previous literature has suggested conflicting results, with Garverick et al. (1976) indicating cattle treated with GnRH had a substantially shorter interval to conception (59.7 ± 9 vs 91 ± 23 days) and required 2.7 fewer days from treatment to first oestrus or ovulation when compared to hCG treatment. Additionally, a recent study has suggested that cattle treated with hCG ovulate significantly later than cattle treated with GnRH, 4.9 hours on average regardless of dioestrus periods (Liu et al., 2019). Moreover, Elmore et al. (1975) found that cattle treated with hCG required 1.9 fewer days from treatment to first oestrus; however, the interval from treatment to conception was 10.4 days longer than their GnRH-treated counterparts. This may be partially due to the fact that hCG-treated cattle, on average, required more breedings per conception than the GnRH-treated group. Henceforth, there is still much debate amongst the scientific community on which treatment induces a more rapid return to cyclicity in dairy cattle afflicted with COD.

It has been suggested that low-circulating progesterone concentrations may have a role in the pathogenesis of COFs due to the role of progesterone in regulating the effects of oestradiol (Wiltbank et al., 2002). Understandably, many publications have investigated luteal profiles (progesterone concentrations and CL diameter), the number of CL present on the ovary, circulating progesterone concentrations, as well as the effects of hCG and GnRH on follicular luteinisation. There was no clear consensus on the effects of hCG and GnRH on the aforementioned parameters. While two articles suggested that there was no significant difference in circulating progesterone concentrations (Yotov et al., 2014 Nakao et al., 1980), another article indicated that cattle treated with hCG (3000 IU) had significantly higher plasma progesterone levels suggesting that hCG may be more effective at luteal phase induction (Singh et al., 2012). Moreover, Singh et al. (2012) found that post-AI luteal profiles were significantly more favourable in hCG treated cattle; it was proposed that this could be the result of elevated pre-conception plasma progesterone concentrations.

The efficacy of GnRH and hCG in treating bovine ovarian follicular cysts should be further evaluated with more controlled studies. Most of the articles found during the literature review for this Knowledge Summary were small studies that did not include a control group receiving no therapeutics in the study design. Further analysis to ensure that spontaneous resolution of ovarian cysts that occur naturally are accounted for in the study design or with statistical modeling would be beneficial; since the rate of spontaneous cure has been reported to be as high as 60% in fresh cows that have not yet ovulated (Ijaz et al., 1987; Jeengar et al., 2014; Kesler and Garverick, 1982). Some articles achieved this by comparing the proportion of animals in each treatment group relative to their days in milk (DIM), suggesting that if proportions of cows less than 40 DIM were equivocal, then the spontaneous cure rates should not be impacting results (Taktaz et al., 2015). Moreover, a network analysis comparing the efficacy of treatment methods may be beneficial. However, due to the heterogeneity of the studies identified, at this time, the treatments are unequivocal, and network analysis cannot be performed (Lean et al., 2009). For example, many studies were excluded as they did not directly compare GnRH and hCG treatments; Nakao et al. (1979) performed a small crossover trial where animals were enrolled in subsequent treatment groups when they failed to respond to previous therapy, making it challenging to differentiate subsequent treatment failures from individual differences in fertility.

To provide more guidance for the clinical recommendation and treatment of ovarian follicular cysts, the route of administration of the chosen therapeutics should also be further investigated. Rizzo et al. (2011) compared the intramuscular and epidural use of lecirelin, a GnRH analogue, to treat bovine ovarian follicular cysts. They found that epidural administration of lecirelin in cows with follicular cysts resulted in a higher percentage of cows returning to oestrus than those cows which received an intramuscular injection of lecirelin (Rizzo et al., 2011). This effect was attributed to the action of lecirelin on GnRH receptors in the spinal cord and ovary, and possibly on the pituitary gland directly through the epidural canal (Rizzo et al., 2011). These findings support the possibility of further improvements in the treatment of bovine ovarian follicular cysts by altering existing treatment methods.

Moreover, studies to determine the safest and most efficacious dose of hCG may also be beneficial. Among the studies in this Knowledge Summary, the dose of hCG administered to cows with ovarian follicular cysts ranged from 1,500 IU (Taktaz et al., 2015) to 3,000 IU (Mollo et al., 2012; Verma & Dabas, 1994; Garverick et al., 1976; Nakao et al., 1992) to as high as 10,000 IU hCG (Elmore et al., 1975). Other reports have published dosages ranging from 500 IU (Yotov et al., 2014 to 20,000 IU (Nakao et al., 1979). Human chorionic gonadotropin has a high molecular weight and carbohydrate content and thus can lead to the production of hCG antibodies and anaphylaxis in cattle (De Rensis et al., 2010; Giordano et al., 2012). To decrease the likelihood of administering an excessive amount of hCG and causing an immune response, the minimum effective dose should be established to guide future treatment plans. Alternatively, treatment with hCG may be divided into multiple smaller doses of hCG, as this has been shown to be equally effective as a single large dose in modulating ovarian follicular development in dairy cows with cystic ovarian follicles (Ono et al., 2018).

As a final note, it was indicated in the literature that hCG, which historically was the treatment of choice for COD and for unknown reasons, became challenging to acquire and cost-prohibitive for producers (Seguin et al., 1976). Seguin et al. (1976) suggested cost increases were due to challenges in obtaining hCG as it is isolated from pregnant women's urine and speculated that the synthetic molecule GnRH would provide a more cost-effective and stable supply chain (Seguin, 1976). Moreover, Ngategize et al. (1987) found that it was more economical to treat cattle with GnRH than with hCG; however, both treatment options were found to be more economically advantageous than no treatment or spontaneous resolution (Ngategize et al., 1987). This conclusion was based on assumptions that GnRH resulted in the more successful resolution of COF, cost less than hCG, but required a longer recovery (Ngategize et al., 1987). Regardless, Ngategize et al. (1987) suggest that days to first oestrus (recovery time) would need to decrease by 50% for hCG to compete out GnRH therapy (Ngategize et al., 1987).

In conclusion, at this time, there is insufficient evidence to suggest whether GnRH or hCG is a more efficacious treatment for ovarian follicular cysts, and further research is required to elucidate which treatment results in a more rapid return to cyclicity for cattle afflicted with cystic ovarian follicles. Without additional evidence when considering practical implementation in field conditions, GnRH is a more appropriate first-line therapeutic for cystic ovarian disease due to challenges surrounding the immunogenicity, availability, and cost of hCG therapy.

## Methodology Section

**Search Strategy:** 

Databases searched and dates covered:	CAB Abstracts database accessed on WOS platform (1973–2021) PubMed Central accessed on NCBI website (1910–2021)
Search strategy:	CAB Abstracts: (dairy OR lactating OR cow OR cattle OR heifer OR bovine) AND ((cystic OR cysts) AND ovarian AND (disease OR follicle OR follicular)) (Reproductive performance OR ovulation OR estrus OR conception OR pregnancy OR preg rate OR oestrus) (human chorionic gonadotropin OR hCG) AND (gonadotropin releasing hormone OR GnRH) 1 AND 2 AND 3 PubMed: (dairy OR lactating OR cow OR cattle OR heifer OR bovine) AND ((cystic OR cysts) AND ovarian AND (disease OR follicle OR follicular)) AND (Reproductive performance OR ovulation OR estrus OR conception OR pregnancy OR preg rate OR oestrus) AND (human chorionic gonadotropin OR hCG) AND (gonadotropin releasing hormone OR GnRH)
Dates searches performed:	20 Dec 2021

**Exclusion / Inclusion Criteria:** 

Exclusion:	Non-English language, non-primary literature, non-bovine, studies not relevant to the PICO question.
Inclusion:	Use of GnRH and hCG for the treatment of ovarian follicular cysts, time as a metric for evaluation.

**Search Outcome:** 

Database	Number of results	Excluded – Non-English language publication	Excluded – Non-primary literature	Excluded – Non-bovine	Excluded – Not relevant to PICO question	Total relevant papers
CAB Abstracts	67	21	17	5	19	5
PubMed	20	4	7	3	5	1
Total relevant papers when duplicates removed						6

## Conflict of Interest

The author declares no conflicts of interest.

The authors are veterinary students at Michigan State University (East Lansing, MI, USA) that completed this Knowledge Summary as part of the LCS 679 Food Animal Production Medicine 1 course.

## Disclaimer

Knowledge Summaries are a peer-reviewed article type which aims to answer a clinical question based on the best available current evidence. It does not override the responsibility of the practitioner. Informed decisions should be made by considering such factors as individual clinical expertise and judgement along with patient’s circumstances and owners’ values. Knowledge Summaries are a resource to help inform and any opinions expressed within the Knowledge Summaries are the author’s own and do not necessarily reflect the view of the RCVS Knowledge. Authors are responsible for the accuracy of the content. While the Editor and Publisher believe that all content herein are in accord with current recommendations and practice at the time of publication, they accept no legal responsibility for any errors or omissions, and make no warranty, express or implied, with respect to material contained within. For further information please refer to our Terms of Use.
